# Etymologia: Streptomycin

**DOI:** 10.3201/eid2503.ET2503

**Published:** 2019-03

**Authors:** Ronnie Henry

**Keywords:** streptomycin, antimicrobial drugs, Selman Waksman, Albert Schatz, Streptomyces griseus, bacteria, soil bacteria

## Streptomycin [strepʺto-miʹsin]

In the late 1930s, Selman Waksman, a soil microbiologist working at the New Jersey Agricultural Station of Rutgers University, began a large-scale program to screen soil bacteria for antimicrobial activity. By 1943, Albert Schatz, a PhD student working in Waksman’s laboratory, had isolated streptomycin from *Streptomyces griseus* (Figure) (from the Greek *strepto*- [“twisted”] + *mykēs* [“fungus”] and the Latin *griseus*, “gray”).

**Figure:**
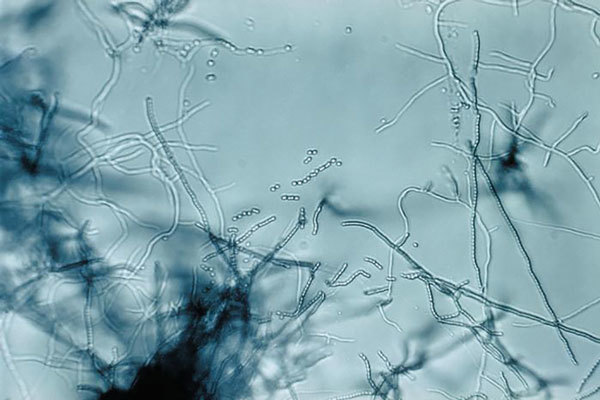
Slide culture of a Streptomyces sp. bacteria, which produces the antibiotic streptomycin. Note the branching filamentous hyphae, abundant aerial mycelia, and long chains of small spores. Image: CDC/Dr. David Berd

In 1944, Willam H. Feldman and H. Corwin Hinshaw at the Mayo Clinic showed its efficacy against *Mycobacterium tuberculosis*. Waksman was awarded a Nobel Prize in 1952 for his discovery of streptomycin, although much of the credit for the discovery has since been ascribed to Schatz. Schatz later successfully sued to be legally recognized as a co-discoverer of streptomycin.
